# Response Surface Optimization of Cactus Pear (*Opuntia ficus-indica*) with Lantana camara (*L. camara*) Fruit Fermentation Process for Quality Wine Production

**DOI:** 10.1155/2020/8647262

**Published:** 2020-01-11

**Authors:** Zenebe Tadesse Tsegay, Solomon Mengistu Lemma

**Affiliations:** ^1^College of Natural and Computational Science, Department of Chemistry, Aksum University, P.O. Box 1010, Aksum, Ethiopia; ^2^College of Dryland Agriculture and Natural Resources, Department of Food Science and Post-Harvest Technology, Mekelle University, P.O. Box 231, Mekelle, Ethiopia; ^3^Department of Chemical and Food Engineering, Bahir Dar Institute of Technology, Bahir Dar University, P.O. Box 26, Bahir Dar, Ethiopia

## Abstract

Fermenting blended fruits has been used to improve fruit wine quality. Cactus pear and Lantana camara fruits have well-known nutritive and health benefits. The purpose of this study was to investigate cactus wine quality improvement by applying response surface optimization method of cactus pear and Lantana camara fruits juice fermentation process. Wine quality responses were optimized at an experimental strategy developed using central composite rotatory design by varying fermentation process variable temperature, inoculum, and Lantana camara fruit juice concentration for six days. The developed fermentation models were significant (*p* < 0.01) to predict alcohol, total phenol content, and sensory property of the final wine accurately. From the statistics calculations, fermentation temperature of 24.8°C, inoculum concentration 10.16% (*v*/*v*), and Lantana camara fruit juice concentration of 10.66% (*v*/*v*) were the overall optimum values to produce cactus pear fruit wine with alcohol 9.53 ± 0.84% (*v*/*v*), total phenol content 651.6 ± 54 (mg L^−1^ equivalent to gallic acid), and sensory value of 8.83 ± 0.29. The Lantana camara fruit juice concentration added had shown significant (*p* < 0.05) enhancement on total phenol content and sensory values of the final wine. The results can be used for large-scale wine production in order to reduce its postharvest losses.

## 1. Introduction

Fermented fruit juice beverages have been developed from a blend of guava with kokum, sapota with ginger (*Zingiber officinale*), watermelon (*Citrullus lanatus*), and mixed fruits (pawpaw and banana). The total quality of these fruit wine can be improved by applying medicinal herbs and other fruits during its fermentation process [1–3]. For instance, the study report by Lee et al. [4] indicated that the total phenol content, anthocyanin content, and brightness were higher in apple wine incorporating with medicinal herb fermentation than in normal apple wine. In addition, Lee and Chen [5] studied on the utilization of ball-milled achenes in strawberry wine fermentation to increase the levels of ellagic acid and total phenol content up to 19.72% and 52.37%, correspondingly. This study reported the antioxidant capacity was enhanced to 54.09% and 51.49% in ABTS and DPPH radical scavenging capacity, respectively. Pawpaw, banana, and watermelon fruits were used to produce mixed fruit wines using *Saccharomyces cerevisiae* with improved physicochemical and sensorial properties of the final wine [1]. Fruits like blueberry, strawberry, and Lantana camara have important nutrients that use to improve wine quality in the fermentation process. Since the nutrient composition of Lantana camara fruit is sufficient in comparison to others like grape and blueberry fruits, it can be used for cactus wine quality enrichment [4, 6].

Cactus pear and Lantana camara fruits are composed of important nutrients which are suitable for wine fermentation substrate. For instance, polyphenols, triterpenes, flavones, and vitamins are present in both fruits [7, 8]. In addition, cactus pear fruits are a basis of nutrients and vitamins and are eaten as fresh, dried, or preserved in jams, syrups, or processed into candy-like products [9]. Most important nutrients such as vitamins, amino acids, minerals, polyphenols, betalains, and indicaxanthin abundantly found in this fruit [10]. According to Inglese et al. [11], data survey orange type of cactus pear fruit contains acidity (% citric acid), soluble solids (°Brix), vitamin C (mg 100 g^−1^), *β*-carotene (mg 100 g^−1^), lutein (*μ*g g^−1^), *β*-cyanins (mgkg^−1^), and *β*-xanthins (mg kg^−1^) is 0.55–0.57, 13.5–14.5, 24.1–28.0, 0.85–2.28, 0.04, 2.4–11.0, and 16.0–76.3, respectively. Inputs of these nutrients and biochemical reactions occurred during fermentation for better wine quality properties were focused in the current study to investigate their impact in the fermentation process.

Lantana camara fruit has also important bioactive compounds that could be applicable for fruit wine quality enhancement. It is a species of flowering plant within the verbena family, *Verbenaceae*. This plant is dominantly grown in most parts of Ethiopia. Its leaf is used as a medicinal plant (Fungi disease treatment) for human in Ethiopia [12, 13]. Children and younger people eat ripe Lantana camara berries. Carstairs et al. [14] reported that ingestion of Lantana camara fruit (including unripe berries) or other its plant parts has no associated negative health effect. Moreover, mature Lantana camara fruit has testy with low acidity properties. From the study reported by Ávila-Reyes et al. [6], about 6.66 mgg^−1^ total flavonoids ascorbic acid equivalent and 14.67 mgg^−1^ gallic acid equivalent of total phenol were present in the ethanol extract of Lantana camara leaf. During this analysis, EC_50_ (half maximal effective concentration) value of this leaf extract has recorded as 3.18 *μ*g mL^−1^ DPPH scavenging effect. These fruit results show its nutritional values could improve wine quality during the wine fermentation process. Therefore, these important bioactive compounds are essential to study its effect on the quality of fruit wine production.

Although cactus pear fruit wine has been reported with its attractive physicochemical and sensorial properties, further enhancement on its sensorial properties is demanded by consumers. Cactus pear (*Opuntia ficus-indica*) fruit wine has physicochemical and sensorial characteristics that are acceptable by consumers [15, 16]. Typically, cactus pear juice from 15.94 7°Brix to 20°Brix fermentable sugar content produces from 5.5% (*v*/*v*) to 9.93% (*v*/*v*) alcohol fermented using *Saccharomyces cerevisiae*. This wine product showed better aroma and flavor perception, volatile compounds, and a velvety feeling of long aftertaste [15, 17]. However, these products are no longer available in limited market due to consumers needed better sensorial quality. Cactus pear fruit wine produced at an optimized fermentation process has acceptable sensory properties [16]. But, further improvement of these fruit wine sensory properties has been suggested by these authors. Moreover, this fruit is a source of nutrients and harnessing of the fruit into a wine product did not get emphasis. Specifically, phenolic bioactive compounds present in Lantana camara fruit could be utilized for wine quality improvement that helps this fruit wine consumers prevent a number of chronic diseases [18].

There is limited study reported on response surface methodology (RSM) optimization of blended fruit fermentation process for better wine quality production. Hence, the current study could be accessible as additional literature data. Likewise, optimizing fermentation process variables of fermentation temperature, inoculum concentration, and blending concentration of Lantana camara fruit juice using RSM could help to develop best models that predict cactus fruit wine qualities. Physicochemical and sensorial properties of cactus fruit wine fermentation process optimization can be achieved by blending Lantana camara fruit juice using RSM. RSM is popular multivariate statistical technique, which has been used in the optimization of many fruit wine fermentation processes [16, 19–25]. RSM is preferable than other one factor at a time optimization techniques. Since one factor at a time is extremely time consuming, expensive to perform larger experimental variables, limited to describe simultaneous effects of dependent and independent variables, RSM is more desirable [26]. RSM is valid for optimizing the processes variables which can be depicted with a second-order polynomial equation. Similarly, RSM coupled with central composite rotatable design (CCRD) uses to select the best fit of polynomial models of fermentation process variables simultaneously [27]. Along with *R*^2^, lack of fit and absolute average deviation (AAD) criteria can be applied to show the suitability of fitted response surface models developed using this statistic technique [28]. Chakraborty et al. [19] used RSM to investigate the quantitative effects of temperature, pH, and duration of fermentation to optimize ethanol content of fruit bael (*Aeglemarmelos L*.) wine. Similarly, flavor compound enhancement of three fermented greengage wines was optimized by controlling fermentation temperature, material to liquid ratio, and sugar content [24]. During the Chinese bayberry and mulberry wine fermentation processes, alcohol, anthocyanin, residual sugar content, total phenolic concentration, total flavonoid concentration, total higher alcohol concentration, and total ester concentration of the final wine were optimized by controlling fermentation temperature, initial sugar content, inoculum size, and initial pH using RSM [20, 23]. Moreover, RSM has been also applied to optimize fermentation conditions for the production of wine from apple and cactus pear fruit to improve its sensory quality [16, 21]. Although, many studies have reported that cactus pear fruit juice can be used for wine production, improving the wine quality by optimizing fermentation conditions with Lantana Camara fruit juice blend was not readily available in the literature. The main objective of this study was to investigate the main effects of prominent process variables on the final wine quality during cactus pear and Lantana camara fruit juice blend fermentation process as well as to determine optimum process conditions using RSM.

## 2. Material and Methods

### 2.1. Materials

Mature cactus pear fruit (variety: *Opuntia ficus-indica*) was obtained from farmers during the peak production time of April and July, Adigrat, Ethiopia. Dark purple colored Lantana camara (*L. camara*) fruits were collected from Axum, Ethiopia, during March and April. Lantana camara (*L. camara*) plant with fruit was previously identified and authenticated by a taxonomist and registered as a voucher specimen number of ET001 at the National Herbarium, College of Natural Sciences and Computation, Addis Ababa University [12].

*Saccharomyces cerevisiae* yeast was purchased from a supermarket, Addis Ababa, Ethiopia, originated by Angel yeast (Hubei, Mainland, China). Sodium phosphate was purchased from Wise Team PLC chemical reagent (Addis Ababa, Ethiopia) originated from UNI-CHEM, India. Ammonium molybdate, sodium molybdate, sodium tungstate, latium sulfate, sodium carbonate, yeast extract, peptone water, D-glucose, phenol (pH value = 4.8‐6.0), potassium dichromate, gallic acid, and sodium thiosulfate were purchased from Wise Team, Addis Ababa, Ethiopia, originated by Loba-Chemie Laboratory Reagents and Fine Chemicals Co. India, Mumbai, India. All chemicals and solvents used in this study were of analytical grade and used as supplied.

### 2.2. Cactus Pear and Lantana camara (*L. camara*) Fruit Juice Extraction and Physicochemical Characterization

The cactus fruits were stored in an icebox at 6°C during half day transportation to Aksum University chemistry laboratory. Using the procedure described by Zenebe et al. [16], 10.5 L cactus pear fruit juice was prepared by a domestic juicer machine (Electric Juicer, BL-727, Japan). Dark purple colored Lantana camara fruits were harvested in clean plastic bags and stored in an icebox at 6°C until arrival at Aksum University chemistry laboratory. First, 1.5 kg of Lantana camara fruits was destemmed, sorted, and washed (twice) by immersing in 3 L distilled water. Then, fruit juice was prepared using domestic juicer (Electric Juicer, BL-727, Japan). During Lantana camara fruit juice extraction, 1 L distilled water was added. To sterilize microbial contamination and prevent unwanted fermentation, 70 mg L^−1^ sodium thiosulfate was added into each juice. Both juices were stored at 4°C until filtered using sterilized cotton cloth mesh to remove the seed and fibers.

Physicochemical characteristics of fresh cactus pear and Lantana camara fruit juices substrate were determined separately before fermentation for further adjustment as well as to compare it with the produced wine as shown in Table 1. The pH values of each fruit juices were determined with a calibrated digital pH meter (model PH-016, Kelilong Electron Co., China). Sugar content was measured using the procedure described by Zenebe et al. [16]. Briefly, 1 mL of each juice samples was diluted into 150 mL distilled water and mixed sufficiently. In the test tubes containing 2 mL standard sugar solution (dextrose glucose) of 5, 10, 25, 50, and 75 mg L^−1^ as well as 2 mL of each fruit juice sample, 1 mL of 5% phenol solution in water (*v*/*v*) and 5 mL of concentrated sulfuric acid were added with sufficient mixing. All the test tubes were allowed to stand for 10 minutes including blank solution contained 2 mL distilled water, 1 mL with 5% phenol solution, and 5 mL of concentrated sulfuric acid, and the solutions were mixed and placed in a water bath at 26°C for 20 minutes. Later, these incubated samples were measured at 490 nm using a UV-visible spectrophotometer instrument (UV-5100 Spectrophotometer, Metash Instruments Co. Ltd., Shanghai, China). Finally, sugar concentrations of juice samples were determined from the calibration equation of absorbance = 0.0126*x* + 0.0175 at *R*^2^ = 0.9989, where *x* stands for sugar concentration (mg L^−1^ D-glucose).

Total phenol contents of samples juice were measured according to the procedure described by Stintzing et al. [29] with some modification. One mL of each juice samples was diluted by 150 mL distilled water. From each dilution, 1 mL was mixed with 5 mL of 10% FC reagent and 4 mL of sodium carbonate solution (7.5%) and incubated at room temperature for 30 min. Standard gallic acid solutions of 10, 25, 50, 100, 150, and 200 mg L^−1^ were prepared to develop a calibration equation. Finally, concentration of total phenols was calculated from absorbance = 0.0117*x* + 0.0188 at *R*^2^ = 0.9995, where *x* represents total concentration of total phenol equivalent to gallic acid (mg L^−1^ EGA). The optical density was measured at 765 nm using spectrophotometer (UV-5100 Spectrophotometer, Metash Instruments Co. Ltd, Shanghai, China) against distilled water as a blank solution.

### 2.3. Fermentation

#### 2.3.1. Inoculum Preparation

The yeast cell (biomass) was prepared. First, 50 mL of sterilized YEPD media (1% (*m*/*v*) yeast extract, 2% (*m*/*v*) peptone, and 2% (*m*/*v*) glucose) was prepared and poured into 150 mL sterilized conical flask. Then, 0.75 g of the dry bakery yeast cell (*Saccharomyces cerevisiae*) (hydrated in 50 mL mild hot distilled water at 35°C) was added into this media and made up to 150 mL using distilled water. Finally, the mixture was incubated in a rotary shaker (VRN-200, Gemmy Industrial Corp. Taiwan) with a speed of 120 rpm at 28°C for 24 hours. Then, it transferred into a 1000 mL Erlenmeyer flask which contained 500 mL of the sterilized cactus pear juice pulp that was previously adjusted at pH of 3.4 using pH meter (model PH-016, Kelilong Electron Co., China). The mixture was incubated at 28°C for 36 hours at a shaker speed of 150 rpm to use it directly for wine fermentation [16].

#### 2.3.2. Fermentation and Stabilization

The final extracted cactus pear juice was adjusted to the sugar content of 200 g L^−1^ (expressed by D-glucose) using table sugar. pH was adjusted to 3.4 using 0.5 g L^−1^ tartaric acid solution. The remaining factors like temperature, inoculum concentration (1.7 × 10^6^ CFU/mL), and Lantana camara fruit juice concentration (70% (*w*/*v*) juice extract) were adjusted according to the final experimental design shown in Table 2. The fermentation process was developed in spherical 500 mL glass flasks that have equal volume and shape contained appropriate volume (350 mL) of adjusted cactus pear juice. The fermentation process was controlled once a day after three days of the fermentation process. The amount of residual sugar present was analyzed during the process by taking a sample from a fermentation process seated at a center point. According to the procedure described by Zenebe et al. [16], absorbance of the fermenting sample was measured at 490 nm using spectrophotometer (UV-5100 Spectrophotometer, China) against the blank solution contained 2 mL distilled water, 1 mL 5% (*m*/*v*) phenol solution and 5 mL of concentrated sulfuric acid (12 × 10^3^ mM). Series standard solutions of 5, 10, 25, 50, and 75 mg L^−1^ dextrose glucose were prepared. Linear equation of absorbance reading = 0.0127*x* + 0.0128 was developed to calculate the residual sugar concentration (x mg L^−1^ D-glucose) at *R*^2^ = 0.997. Plastic valve airlocks were used on stationery fermenters for proper venting till the fermentation finished within 6 days. Free run of the fermented products was filtered from each sample run using sterilized cotton cheese close. Each sample was packaged in a sterilized brown 330 mL glass bottles. Finally, each wine sample was pasteurized at a temperature of 65°C for 20 minutes and preserved for twenty days at room temperature (24°C).

### 2.4. Experimental Design and Data Investigation

#### 2.4.1. Experimental Uncertainty

Total uncertainties of the predicted values shown in Table 3 were determined using the following equation [30].

(1) $$\begin{array}{c} U_{x}=\frac{{SE}_{YX}}{m}\text{ x }\sqrt{\frac{1}{k}+\frac{1}{n}+\frac{{\left(Y_{x}-Y_{\text{avg}}\right)}^{2}}{m^{2}\text{ x }{\left(n-1\right)}^{2}\text{ x }{\text{S}}_{x}^{2}}}, \end{array}$$

where *U*_*x*_, *SE*_*Yx*_, *Y*_*x*_, *Y*_avg_, *k*, *m*, *n*, and *S*_*x*_ stand for the standard uncertainty of the concentration of the analyte measured, standard error regression of the linear equation, average value of the “*k*” replicate measurements, average signal for the calibration standards, number of replicates used to establish the sample's average signal, slope, number of calibration standards, and variance of the calibration standard, respectively.

The interactive effects of fermentation temperature, yeast inoculum concentration, and concentration of Lantana camara fruit juice on alcohol production, polyphenol content, and sensory score value of the fruit wine were analyzed using the multivariate statistical optimizing technique, that is response surface methodology (RSM). As it can be seen in Table 4, central composite rotatable design (CCRD) for 3 parameters that has 6 axial points (± *α*), 6 center, and 8 factorial points was used to develop 20 experimental runs. To fit experimental data, to determine critical points (maximum, minimum, or saddle), and to understand impacts of independent fermentation variables on responses, polynomial function (Equation (2)) was used.

In the control fermentation process for optimizing lantana camara fruit concentration, the control fermentation process for optimizing lantana camara fruit concentration was carried at fixed pH (3.9), inoculum concentration (16%, *v*/*v*), and temperature 30°C as reported by Zenebe et al. [16].

In the control fermentation process for optimizing temperature and inoculum concentration, the control fermentation process for optimizing temperature and inoculum concentration was tested at different lantana camara fruit concentrations (%, *v*/*v*), namely, 6, 8, 10, 12, and 14. The smaller concentration (6%, *v*/*v*) did not affect and the largest concentration (%, *v*/*v*) shown a darkening effect on the prepared wine. Therefore, 10% (*v*/*v*) Lantana camara fruit concentration was selected as the best control.

(2) $$\begin{array}{c} Y=\beta_{0}+\overset{3}{\underset{i=1}{\sum }}\beta_{i}x_{i}+\overset{3}{\underset{i=1}{\sum }}\beta_{ii}x_{i}^{2}+\overset{3}{\underset{i=1}{\sum }}\overset{3}{\underset{j=\left(i+1\right)}{\sum }}\beta_{ij}x_{i}x_{j,} \end{array}$$

where *Y* stands for those responses to be predicted (alcohol content, total phenol content, and sensory score value of the fruit wine); *i* and *j* represent linear and quadratic coefficients, correspondingly; *x*_*i*_ and *x*_*j*_ correspond to three independent variables: fermentation temperature, inoculum concentration, and Lantana camara fruit concentration; *β*_0_ (intercept), *β*_*i*_ (linear effects), *β*_*ii*_ (squared effects), and *β*_*ij*_ (interaction terms) were for regression coefficients.

Experimental results were measured at triplicate and mean values nearest to two digits were reported. Response surface analysis of data was displayed by 3D surface and counterplot methods, by keeping one variable constant at the center point (constant) and varying the other variables within the experimental range to show the stationary points (maximum, minimum, or saddle).

More than one variable at a time analysis of variance (ANOVA) were used to evaluate the validity of the models developed using statistical software packages Design Expert version 10.0.3 (Stat-Ease Inc., Minneapolis, MN, USA). Moreover, model adequacies were tested by AAD, *R*^2^, adj-*R*^2^, pre-*R*^2^, adequacy of precision, lack of fit, and C.V. Coefficients of determination (*R*^2^) and absolute average deviation (AAD) were used to analyze the overall predictive capabilities of models developed using this software. Absolute average deviation (AAD) of fitted models was calculated using Equation (3) [28]. Simultaneous optimizations of the multiple responses were carried using a numerical optimization technique by the Design Expert software. Desired goals of each variable and responses were chosen concerning alcohol content, polyphenols, and sensorial values to be maximized. To validate the optimum combination of those three independent parameters, three confirmatory experiments were performed under the optimum fermentation conditions. Significance of the difference between confirmatory experiment and predicted values was tested using the *t*-test (*α* = 0.05). Investigating the adequacy of experimental data is quantified by

(3) $$\begin{array}{c} \text{AAD}=\left\{\frac{\left[\sum_{i=1}^{p}\left(\left(\left|y_{i\exp}-y_{i\text{cal}}\right|\right)/y_{i\exp}\right)\right]}{p}\right\}\text{x }100, \end{array}$$

where *p*, *y*_*i*exp_, and *y*_*i*cal_ represent the number of experiment, experimental, and calculated responses, respectively.

### 2.5. Physicochemical Analysis of the Wine

#### 2.5.1. Estimation of Ethanol

Ethanol (%, *v*/*v*) concentrations of wine samples were determined according to the procedure described by Babu et al. [31] using acidified dichromate solution. The distillation process was carried according to the method used by Zenebe et al. [16]. Briefly, 30 mL distilled water and 1 mL of wine sample mixed together into 50 mL receiving flask, 25 mL of potassium dichromate solution (0.17 M K_2_Cr_2_O_7_ dissolved in 5.9 M H_2_SO_4_) was poured in the mixture. Then, 20 mL of the distillate samples was collected and incubated in a water bath heated at a temperature of 62.5°C for 20 minutes from the mixed samples. Finally, cooled at room temperature and 5 mL of each solution was measured its absorbance at 600 nm. Standard ethanol solutions of 2, 6, 8, 12, and 14 (%, *v*/*v*) were prepared in distilled water. Finally, alcohol concentrations were determined using the calibration equation of absorbance reading = 0.069*x* − 0.0216 at *R*^2^ = 0.998, where *x* indicates ethanol concentration (%, *v*/*v*).

#### 2.5.2. Total Phenol

Total phenol contents of the produced wine samples were analyzed according to the procedure described by Arrizon et al. [32] with some modifications. One mL of each wine samples was diluted into 150 mL distilled water. To develop calibration equation, standard gallic acid solutions of 10, 25, 50, 100, 150, and 200 mgL^−1^ were prepared in distilled water. One mL of each diluted wine samples, standards and blank (distilled water) were mixed with 5 mL of 10% Folin-Ciocalteu's reagent and 4 mL of 7.5% Na_2_CO_3_ solution in 10 mL test tubes. Finally, all the samples and standard as well as the blank solutions were incubated for 16 minutes at temperature 30°C in water bath with test tubes covered and sealed by aluminum foil. The spectrophotometer absorbance of each sample was measured at 765 nm. Finally, concentrations of total phenol in each wine samples were determined from the calibration equation: absorbance reading = 0.082*x* + 0.036 at *R*^2^ = 0.999, where *x* represents for total phenol concentration (mg L^−1^ EGA), respectively.

#### 2.5.3. Sensory Evaluation

Five female and five males trained to evaluate the wine sensory quality. Accordingly, they allowed evaluating the overall sensorial acceptability of the produced wine based on the evaluation score of the 9-point hedonic scale, where nine denotes excellent and one very poor. Randomly distributed 30 mL wine samples filled into150 mL tulip-shaped wine glasses (covered with watch glass) were evaluated by panelist. According to the developed experimental design, two wine samples were examined by each panelist including the replicate samples. Each panelist evaluated every wine sample three times for color, taste, flavor, and overall acceptability on the nine-point hedonic scale (1 = dislike extremely, 2 = dislike very much, 3 = dislike moderately, 4 = dislike slightly, 5 = dislike, 6 = neither like nor dislike, 7 = like slightly, 8 = like, and 9 = like extremely). Evaluation condition was developed as the procedure described by Zenebe et al. [16].

## 3. Results and Discussion

### 3.1. Fitting the Response Surface Models

In the results for three independents, fermentation temperature, inoculum concentration, and Lantana camara fruit juice concentration, variables of the CCRD experimental design and analysis are shown in Table 4. Each of the responses variables was analyzed using second-order polynomial response surface models shown in Equations (4), (5), and (6) and calculated predicted values are presented in Table 4. The predicted optimum levels of fermentation temperature, inoculum concentration, and Lantana camara fruit juice concentration were obtained by applying regression analysis to Equations (4), (5), and (6) using Design Expert software.

To examine the statistical significance of factors and model, analysis of variance (ANOVA) and regression analysis were conducted as shown in Table 5. Moreover, Table 5 shows the estimated regression coefficients of the quadratic polynomial models for the response variables, along with the corresponding coefficients of determination (*R*^2^). Adequacy of models was checked by absolute average deviation (AAD), pre-*R*^2^, adequacy of precision, PRESS, and adj-*R*^2^ as shown in Table 6.

Insignificant lack of fit of models representing the experimental data is indicated by a high probability value [27]. Response predictor with a significant lack of fit which could be indicated by a low probability value was discarded. As shown in Table 5, none of the response predictors depicted in a significant *p* value for selected variables which shows all models have not significant lack of fit. Therefore, these models were sufficiently accurate for predicting the relevant responses of the fermentation process. As it can be seen in Table 5, the developed model Equations (4), (5), and (6) have *F* values of 6.76, 16.3, and 4.96, respectively, which indicates the models were very significant (*p* < 0.01) and there were only 0.31%, 0.01%, and 0.99% chance that the models *F* value this large could occur due to noise correspondingly. This implies that the developed model equations are sufficiently accurate to predict the quality predictive responses of the fermentation process.

The proportion of variation in the response attributed to the model rather than to random error is expressed by the coefficient of determination (*R*^2^). Similarly, coefficient of determination not less than 80% suggests as the model is well fitted [27]. As can be seen in Table 6, all the models have *R*^2^ greater than 80%. Significance of the suitability of fitting the empirical model to the actual data is indicated when *R*^2^ approaches to unity [21]. Since *R*^2^ index is a measure for the amount of the decreasing changeability of response achieved by using the repressor variables in the model as well as increasing a variable to the model will increase *R*^2^ without considering the statistical significance of the additional variable; *R*^2^ index alone cannot demonstrate the accuracy of the model. Hence, for a better measure of accuracy, summary statistic of statistical dispersion or variability from a central point should be calculated using the absolute average deviation (AAD) (Equation (3)). Furthermore, AAD between the estimated and observed data must be as low as possible and suitable values of *R*^2^ and AAD imply that the fitted model depicts the correct behavior of the process and it can be successfully used for the optimization of the wine fermentation processes [28]. The absolute average deviations of alcohol content, total phenol content, and sensory analysis of the produced wine are shown in Table 6.

(4) $$\begin{array}{c} \text{Alc}\left(\%,\frac{v}{v}\right)=58.982.15\text{A}+3.72\text{B}+4.27\text{C}-0.04{\text{A}}^{2}-0.17{\text{B}}^{2}-0.21\;{\text{C}}^{2}, \end{array}$$

(5) $$\begin{array}{c} \text{TP}\left(\text{mg }{\text{L}}^{-1}\right)=-5290.23+165.95\text{A}+432.68\text{B}+330.63\text{C}+14.26\text{BC}-3.43{\text{A}}^{2}-28.95\;{\text{B}}^{2}-22.36\;{\text{C}}^{2}, \end{array}$$

(6) $$\begin{array}{c} \text{Sensory}=-45.31+1.73\text{A}+4.07\text{B}+2.08\text{C}+0.02\text{ AB}-0.04{\text{A}}^{2}-0.20{\text{B}}^{2}-0.12{\text{C}}^{2}, \end{array}$$

where A, B, C, Alc, and TP stand for temperature, inoculum concentration, Lantana camara fruit concentration, alcohol content, and total phenol, respectively.

### 3.2. Effects of the Fermentation Conditions on Responses

**Figure 1:**
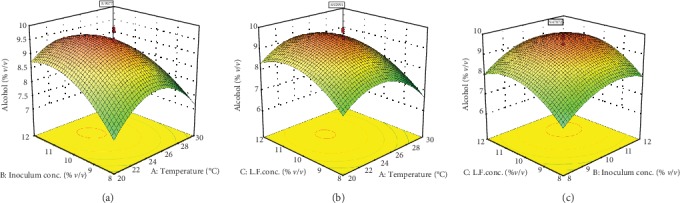
Response surface and contour plots for the effect of inoculum concentration (a, Lantana camara fruit concentration = 10%*v*/*v*) and temperature; Lantana camara fruit concentration (b, inoculum concentration = 10%*v*/*v*) and temperature; Lantana camara fruit concentration (c, temperature = 25°C) and inoculum concentration on the alcohol content of the produced wine.

**Figure 2:**
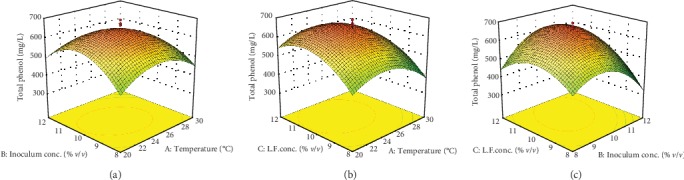
Response surface and contour plots for the effect of inoculum concentration (a, Lantana camara fruit concentration = 10%*v*/*v*) and temperature; Lantana camara fruit concentration (b, inoculum concentration = 10%*v*/*v*) and temperature; Lantana camara fruit concentration (c, temperature = 25°C) and inoculum concentration on the total phenol content of the produced wine.

**Figure 3:**
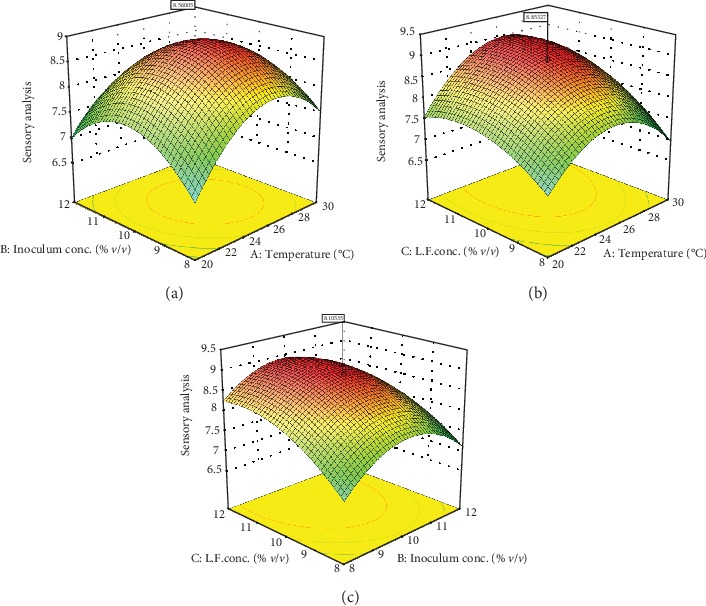
Response surface and contour plots for the effect of inoculum concentration (a, Lantana camara fruit concentration = 10%*v*/*v*) and temperature; Lantana camara fruit concentration (b, inoculum concentration = 10%*v*/*v*) and temperature; Lantana camara fruit concentration (c, temperature = 25°C) and inoculum concentration on the sensory value of the produced wine.

Linear, interactive, and quadratic effects of independent fermentation process variables on alcohol content, total phenol content, and sensory value of the produced wine samples were checked by the significance of every coefficient at *p* values (Table 5). Similarly, mutual interactions among the tested variables are understood by *p* values. Model terms which do not have significant effect (*p* > 0.1) on the response variables and can damage model equations (Equations (4), (5), and (6)) were rejected. Response surface and contour plots shown in Figures 1–3 were generated using Design Expert software to visualize the combined effects of two factors on any response. The visualization of each fitted models was carried as the function of two independent variables while keeping the other variable at the central value. As it can be seen in Table 5, the interaction effect of fermentation temperature and Lantana camara fruit juice concentration has shown insignificant effect (*p* > 0.1) on alcohol content, total phenol, and sensory properties of the final wine.

#### 3.2.1. Alcohol Content of the Produced Wine

From the fermentation process model for alcohol content, the linear and quadratic effects of inoculum and of Lantana camara fruit juice concentrations were significant (*p* < 0.05) as shown in Table 5. Although the fermentation temperature of the linear effect was not significant (*p* > 0.1), the coefficient of quadratic effects was significant (*p* < 0.0001). Generally, the effects of all quadratic terms of independent factors on alcohol content were explained by more than 99% followed by linear effects of inoculum concentration and concentration of Lantana camara fruit juice (95%). All interaction terms of the fermentation process variables have shown insignificant effect (*p* > 0.1) to the alcohol content of the produced wine. Linear and quadratic effects of the inoculum concentration on the alcohol content of the final wine are significant (*p* < 0.05). As it can be seen in Figure 1, initial increase in inoculum concentration rapidly improved the alcohol content. But further increase reduced the alcohol content to about 8% (*v*/*v*) which is similar observation with the study reported by Du et al. [20]. The main reason to reduce alcohol production with an increase in inoculum concentration is growth and reproduction of yeast cells is dependent on the initial nutritional content of the cactus pear and Lantana camara fruit juice. Hence, the metabolic reaction of the fermentation process is progressively decreased upon consumption of nutrient and more CO_2_ is released which suppress the alcohol production [33]. The insignificant interaction effect of fermentation temperature and inoculum concentration on ethanol content is consistent with study reported on blueberry and mango (*Mangifera indica L*.) fruit juice fermentation for wine production [22, 25].

#### 3.2.2. Total Phenol Content of the Produced Wine

The result for the effect of fermentation parameters in Table 5 indicates that linear and interaction effects of fermentation temperature and Lantana camara fruit juice concentration were significant (*p* < 0.1). But, linear, interaction effects of the inoculum concentration with fermentation temperature and Lantana camara fruit juice concentration were insignificant (*p* > 0.1). High significant (*p* < 0.0001) effect by all quadratic terms of the fermentation process was observed on the total phenol content of the wine. The linear effect of fermentation temperature on total phenol concentration enhancement is due to increase in fermentation temperature facilitates solubility rate and diffusion of phenolic compounds during fermentation which is consistent with the study reported by Tchabo et al. [23]. Moreover, the significant effects (*p* < 0.1) of quadratic, interaction of inoculum concentration with Lantana camara fruit juice revealed that total phenol concentration improved due to yeast can convert nonphenolic compounds into phenolic compounds. Moreover, linear, interaction, and quadratic effects of Lantana camara fruit juice concentration have shown a significant effect (*p* < 0.1) on total phenol content of the wine. This is due to the fact that high total phenol concentration in Lantana camara fruit juice concentration directly involved in increasing the total phenol content of the final wine. In addition to this, hydrolytic enzymes such as esterases could be discharged soluble conjugated or insoluble bound phenolic acids by yeast from the cell walls of the cactus pear and Lantana camara fruit juices [23].

Total phenols of single fruit juice concentration are decreased during fermentation process due to biochemical changes. During Carissa spinarum fruit juice wine fermentation process, considerable total phenol content was decreased (from 162.2 mg GAE/100 mL of the fruit juice into 134.9 mg GAE/100 mL of the final wine) [34]. Polyphenol concentration of the final wine can be affected due to many intrinsic and extrinsic factors of the fermentation process. During the fermentation process, reactions such as condensation and polymerization as well as adsorption of polyphenols onto yeast cell wall can be occurred which are the main reason for decreasing total polyphenols in the final wine. Fruits with lower total phenol are subjected to decreasing polyphenol concentration during the fermentation process. Lantana camara fruit has lower pulp content as compared to cactus pear fruit and cannot be used accessibly for wine production. But, this fruit has considerable total phenol content and antioxidant properties [35]. Therefore, adding fruits like Lantana camara with larger polyphenol concentration during the fermentation process can be improved total phenol concentration of the final wine. Similar findings were reported by Lee et al. [4] on total phenolic content of apple wine was improved by fermenting apple fruit juice incorporated with pine needles and medical herbs (hwanggi and mistletoe) the so-called “apple-pine” and “apple-herb.”

#### 3.2.3. Sensory Property of the Produced Wine

Table 5 reveals that the linear effects of fermentation temperature and Lantana camara fruit juice concentration had significant (*p* < 0.05) effect on the sensory value of the final wine. All coefficients of quadratic terms in Equation (6) showed significant influence on the sensory property (*p* < 0.0001) which is consistent with the study reported by Zenebe et al. [16]. In addition, the interaction of fermentation temperature and Lantana camara fruit juice concentration showed significant effect (*p* < 0.1). Origin, fruit type, and fermentation techniques are mainly determinant factors to the complexity of the wine sensory properties. Aroma profiles and concentration of fruit wine are influenced by fermentation temperature. Higher alcohols such as isoamyl alcohol and phenylethyl alcohol are produced in the range of fermentation temperature 15 to 30°C. Besides, esters are the main contributors of fruity aroma and sensory character of wine which are significantly influenced by fermentation temperature [36]. The reason for the change in the sensory property of wine could be due to fermentation temperature-enhanced biomolecules solubility from the fermenting substrate. Volatile organic compounds released from yeast cells and substrates during fermentation are also responsible for sensory acceptance and rejection of fruit wine. The fruity and floral aroma of fruit wine is produced due to the presence of *α*-terpineol and other terpenoids. Moreover, organic compounds like ethyl acetate and acetaldehyde produced during fermentation influence final wine to have fruity and a fermented aroma correspondingly, while acetic acid is not a required aroma in alcoholic beverages since it is considered a product of a poorly controlled fermentation process [32].

Significant effects (*p* < 0.0001) of both linear and quadratic terms of Lantana camara fruit juice concentration (Table 5) show improvement in sensory property of cactus pear fruit wine. In the previous cactus pear fruit wine production study, sensory acceptance value was reported to about 8 [16]. In the current study, it has improved to about 8.7 sensory values. Flavonoid and phenolic compounds present in Lantana camara plant are responsible for the improvement of the current wine sensory property [37]. Lee et al. [4] reported that apple wine produced by supplementing medicinal herbs has shown a better preference in taste, color, and flavor with the normal apple wine. Moreover, adding ball-milled achenes during strawberry wine fermentation causes astringency and harshness in the final wine [5]. Therefore, this study provides an opportunity to improve sensory properties of cactus pear wine by supplementing Lantana camara fruit juice which has nutrients, minerals, vitamins, aroma, and taste accessible to consumers by fermenting them into wine product [38].

### 3.3. Optimization of the Fermentation Process

Three-dimensional representations of the response surfaces (Figures 1to 3) generated by the model coefficients presented in Table 5 were used to indicate the maximum or minimum values of the independent and dependent fermentation variables of the final wine alcohol content. Within the experimental range given in Table 2, data were generated by varying two variables and keeping one variable at the center value of the testing range (control level). Smallest ellipse in the contour diagram of the three-dimensional diagram in Figure 1 shows the maximum predicted value of the produced alcohol. Furthermore, Figure 1 shows the alcohol content of the produced wine with the variation of independent fermentation variables and their respective center value of the testing ranges. The three-dimensional surface plot (Figure 1(a)) of the fermentation process depicts alcohol content was produced exponentially as the concentration of yeast inoculum enlarged. Fermentation temperature facilitated the fermentation process to about 25°C, but further increased suppressed alcohol production. It is obvious that the fermentation process is temperature dependent since yeast biomass declined during the fermentation process when the temperature increased. Although the optimum temperature of *Saccharomyces cerevisiae* yeast in the previous wine fermentation process was recorded at about 29°C, suitable fermentation temperature of the current wine fermentation process to produce optimum alcohol (8.97%, *v*/*v*) was recorded at 25°C [16]. As it can be seen in Figure 1(c), both increased concentrations of inoculum and Lantana camara fruit juice during the fermentation improved alcohol production exponentially. Initially, about 55.47 g L^−1^ of sugar was present in the Lantana camara fruit juice before fermentation. As a result, further production of alcohol to about 9.47% (*v*/*v*) during the fermentation process was observed. Furthermore, more important biochemical nutrients could be added to the substrate by adding further Lantana camara fruit juice concentration which facilitated the fermentation process to increase the alcohol content. Production of alcohol at the early phase of the fermentation process is very fast due to the consumption rate of sugars by yeasts at the fast growth phase. Further addition of inoculum and Lantana camara fruit juice concentrations could no longer increase alcohol production due to yeast cells are suppressed by the produced alcohol [4]. Rapid release of CO_2_ is another factor of fermentation temperature to produce less alcohol content. This is due to the fact that more CO_2_ suppress yeast cell growth which is also reported by Du et al. [20]. Increasing fermentation temperature for Chinese bayberry wine production from 24 up to 28°C favored to produce more alcohol but started to decline when the temperature was greater than 28°C [20]. To sum up, the optimum fermentation temperature, inoculum concentration, and Lantana camara fruit juice concentration to produce the final wine with an alcohol content of 9.9% (*v*/*v*) were 25°C, 10% (*v*/*v*), and 10% (*v*/*v*), respectively.

In the current study, the amount of alcohol produced is about 10% (*v*/*v*) which is lower than most commercial fruit wines should contain. Therefore, from the health perspective, wine with lower alcohol content can have more consumer acceptance than higher alcohol to get required nutrients. Hence, the produced wine could be attractive by consumers since it has a lower alcohol content as compared to wine with alcohol content range from 11 to 15% (*v*/*v*) [39].

Figure 2 shows the response contour and surface plot visualization of the predicted model equation for the total phenol content of produced wine. The exponential increased in inoculum concentration and fermentation temperature on the total phenol revealed in Figure 2(c) suggests that solubility rate and diffusion of phenolic compounds were enhanced. Esterase induced hydrolysis of esters into phenolic acids occurred during the fermentation process is also another effect of booth factors which is similar to the study reported by Tchabo et al. [23]. In addition, acid hydrolysis and bioconversion of condensed phenolic compounds present in cactus pear fruit juice during fermentation in the presence of yeast cells increased total phenol concentration [10]. The maximum total phenol content recorded at optimum fermentation temperature (25°C) and inoculum concentration (10%, *v*/*v*) at constant Lantana fruit juice concentration of 10% (*v*/*v*) was about 643.1 mg L^−1^. As can be seen in Figure 2(b) total phenol concentration increased slightly with increasing temperature at about 24.5°C and declined at about 27°C. Conversely, total phenol content increased with the exponential increase in Lantana camara fruit juice concentration. At an optimum temperature (24°C), inoculum concentration (10%, *v*/*v*) and Lantana camara fruit juice concentration (10%, *v*/*v*) about 643 mg L^−1^ total phenol concentrations were measured. Total phenol content of the final wine exponentially increased to about 644 mg L^−1^ with increased Lantana camara fruit juice concentration and semiexponentially increased with increased inoculum concentration which is indicated in Figure 2(c).

Optimized fermentation process parameters and their interaction effects on the final wine sensory quality were visualized using response surface plots. Specifically, response surface plots shown in Figure 3 are the visualization of the sensory quality predicting model equation (Equation (6)). Moreover, quadratic response surface plots shown in Figure 3 illustrate the wine sensory quality profile in the optimization of two fermentation parameters by keeping the third variable at control (center) point. As it is shown in Figure 3(a), wine sensory quality profile was increased exponentially with the increase in temperature up to 28°C and declined above 29°C. Similarly, increasing inoculum concentration about 10% (*v*/*v*) enhanced the wine sensory quality profile on the contrary the quality decreased the inoculum below 10%. As can be seen in Figures 3(b) and 3(c), wine sensory quality score was increased gradually with an initial increase in Lantana camara fruit juice concentration to about 11.5% (*v*/*v*). Furthermore, wine sensory quality score was exponentially increased due to the interaction effect of Lantana camara fruit juice concentration with fermentation temperature and inoculum concentration. The optimum fermentation process to produce maximum sensory quality score value 8.9 was at fermentation temperature of 25°C, inoculum concentration of 10% (*v*/*v*), and Lantana camara fruit juice concentration of 10% (*v*/*v*).

The maximum and minimum levels of the fermentation variables in quadratic models shown in Equations (4), (5), and (6) were calculated through the first derivate of these mathematical functions, which describes the response surface and equates it to zero [26]. Finally, the predicted optimum levels of the fermentation temperature, inoculum concentration, and Lantana camara fruit concentration were obtained from the linear equations of the first derivate of these mathematical functions. The predicted and experimental alcohol, total phenol content, and sensory properties of the fermentation process were also determined as shown in Table 7. All the predicted levels of the fermentation variables lay in the range of the developed experimental design (Table 2). The Student *t*-test (*t*_0.1,2_) for the predicted and measured response of alcohol content, total phenol, and sensory value was calculated as 2.57, 2.72, and 2.01. There is insignificant (at 90% confidence level) difference between the experimental and predicted response values. In the optimization study, the optimum fermentation variables for higher alcohol content can be obtained at 26.88°C, 10.94% (*v*/*v*) inoculum, and 10.17% (*v*/*v*) Lantana camara juice. At these optimum fermentation variables, the maximum alcohol content of the final wine was measured as 7.46% (*v*/*v*).

### 3.4. Multiresponse Optimization of Fermentation Process Variables and Verification of Results

**Figure 4:**
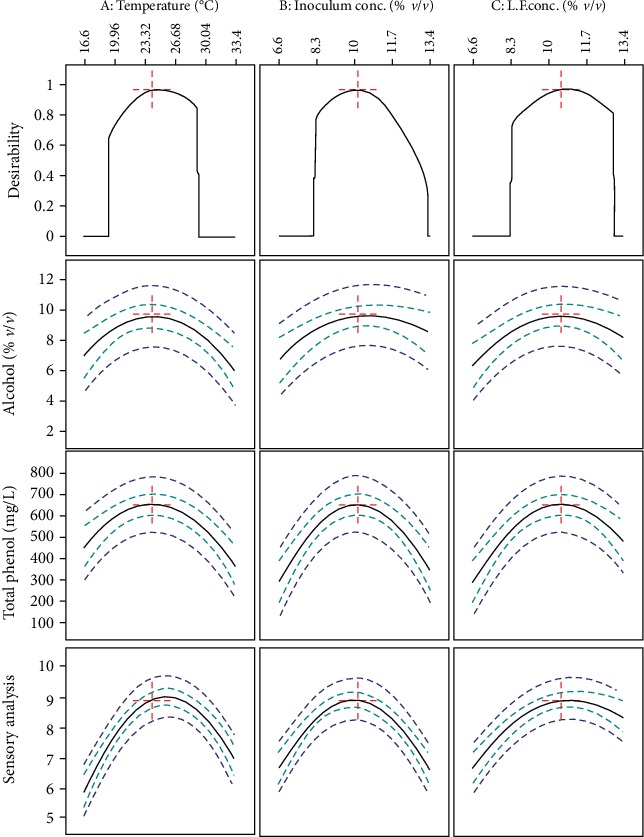
Numerical multiresponse optimization and desirability value (the numbered buttons) across the top correspond to the solution list. Units are temperature in °C, inoculum and Lantana camara fruit concentration in %, *v*/*v*.

Desirability function approach is applicable for optimization of multiple response by converting first the response into an individual desirability function that varies from lowest desirability to highest desirability (from 0 to 1). Then, the partial desirability functions are combined into a single composite response, which is called an overall desirability function [40]. In the current study, one-sided transformation using Design Expert software was applied to maximize those three response variables for selecting higher and the better response. From the fermentation processing variables that maximize alcohol content, total phenol content, and the most sensory acceptability were selected. The overall desirability (0.936) of optimization was found to be very high which is required. Results of optimized processing temperature, inoculum concentration, and Lantana camara fruit concentration at 0.936 desirability function were 24.8°C, 10.16% (*v*/*v*), and 10.66% (*v*/*v*), respectively, as shown in Figure 4. At these values predicted alcohol, total phenol content, and sensory value were 9.53 ± 0.84% (*v*/*v*), 651.6 ± 54 (mg L^−1^ equivalent to gallic acid), and 8.83 ± 0.29, respectively. The predicted overall optimum alcohol content of the final wine is almost equivalent to the previous reports on cactus pear fruit fermentation [15, 16]. The overall predicted response for sensory value was improved from 7.74 to about 8.83 due to the addition of Lantana camara fruit juice during the fermentation process [16]. Initial total phenol content of the fermentation substrate (cactus pear fruit juice) was 332.6 ± 16.3 (mg L^−1^ equivalent to gallic acid). Due to biochemical reaction and the addition of Lantana camara fruit juice during the fermentation process, overall predicted total phenol content was enriched about 651.6 ± 54 (mg L^−1^ equivalent to gallic acid) which is similar to the study reported on apple wine total phenol enhanced due to the addition of medicinal herbs [4]. Figure 4 shows numerical multiresponse optimization of all fermentation variables as well as desirability constant along with all responses.

Predicted optimum fermentation conditions should be confirmed by comparing the measured responses with the responses predicted through the model [28]. Triplicate measurement of response under the predicted optimum conditions (fermentation temperature = 24.8°C, inoculum concentration = 10.16%(*v*/*v*), and Lantana camara fruit juice concentration = 10.66%(*v*/*v*)) was carried. As it can be seen in Table 8, all mean values of measured responses are in the range of the predictive interval (95%) which shows response values perfectly matched the predicted response values. This table also reveals that using the Student *t*-test (*t*_0.05,2_), the difference between the measured responses and the responses predicted through the model is too small to be explained by indeterminate sources of errors which suggests that model equations are not affected by the determinate source of errors. In general, developed response surface equations had a high capability to determine the quality predictive responses of the fermentation process.

### 3.5. Validation of Developed Quality Predictive Models

All the response variables shown in Table 6 have *R*^2^ higher than 0.80, indicating the regression models were suitable to explain the fermentation behavior. The total deviation of responses models for alcohol content, total phenol content, and sensory analysis in the fermentation process are explained by 82%, 92%, and 96%, respectively. This percentage of the coefficient of variation (*R*^2^) implies good agreement between the experimental and predicted response values of the wine production. Moreover, AAD values shown in Table 6 indicate only 0.95%, 2.696%, and 1.17% of deviation between the estimated and observed data of the response models for alcohol content, total phenol content, and sensory analysis during the wine fermentation process, respectively. Suitable values of *R*^2^ and AAD imply that the fitted models depict the correct behaviors of the fermentation process.

Standard deviation, mean value, coefficient of variation (CV), adj-*R*^2^, pre-*R*^2^, adequacy of precision, and PRESS of all developed response models are also shown in Table 6. It is expected from an adequate model that (Adj-*R*^2^–Pre-*R*^2^) should be less than 0.2; maximum PRESS; Pre − *R*^2^ > 0.7; and adequacy precision > 4 [27]. As it can be shown in Table 6, except for response for alcohol content has (Adj-*R*^2^–Pre-*R*^2^) slightly greater than 0.2 (0.4) and Pre − R^2^ < 0.7 (0.34), both responses for total phenol content and sensory value have (Adj − *R*^2^–Pre − *R*^2^) < 0.2 and Pre − *R*^2^ > 0.7.Moreover, all the responses indicated maximum PRESS and adequacy precision > 4. The validity of the optimal simultaneously predicted fermentation responses was tested by conducting three confirmatory experiments under the overall optimized combination input fermentation variables which are shown in Table 8.

### 3.6. Limitation of the Study

In the current fermentation process, addition of supplementary nutrients (limiting factors) such as nitrogen or some traceable elements content was not considered. The absence of these nutrients in effect in the fermentation process and final wine quality will be the main topic for further study. Specific types of *Saccharomyces cerevisiae* yeast strain, amount of and type of glucose added, and both fruits' origin require further optimization for better quality of the fruit wine. Moreover, adding pectolytic enzymes during juice extraction may have enhanced solubilization of nutrients from the cactus and Lantana camara for better fermentation yield. The drawback, these enzymes are responsible for the production of considerable methanol on the final wine product which increases toxicity. Hence, further fermentation optimization that considers the abovementioned factors may address the limitation.

## 4. Conclusion

In this study, cactus pear and Lantana camara fruits, juice fermentation process optimization by applying response surface methodology for quality wine production was investigated. Accordingly, cactus pear fruit wine that has improved total phenol and the better sensory property was developed. The developed fermentation process predictive models have shown significant adequacy (*p* < 0.01) and insignificant lack of fit to predict alcohol content, total phenol content, and better sensory properties of the produced wine. There are only 0.95%, 2.70%, and 1.17% of absolute average of deviation (AAD) between the data that experimentally measured and calculated using the fitted models. Linear and quadratic terms of the Lantana camara fruit juice concentration have shown significant (*p* < 0.05) effects on alcohol, total phenol, and sensory acceptance during the fermentation process. Adding Lantana camara fruit juice concentration during the fermentation process has enhanced the total phenol content and sensory values of the cactus fruit wine due to adequate bioactive compounds availability in this fruit. In general, fruits like Lantana camara could be used for wine quality improvement especially fruit wine produced from fruits with sufficient nutrient but lower polyphenol content and sensory acceptance in wine production. Medicinal application of the produced wine at the optimized or at further blending ratio of the fruits fermentation process needs further studies.

## Figures and Tables

**Table 1:** Physicochemical characteristics of fresh cactus pear and Lantana camara fruit juices.

Parameter	Cactus pear fruit juices	Lantana camara fruit juices
pH	5.86 ± 0.035	5.43 ± 0.15
Sugar content (g L^−1^ D-glucose)	93.79 ± 1.27	55.47 ± 0.93
Total phenol (mg L^−1^ gallic acid)	332.6 ± 16.3	578.2 ± 23.5

**Table 2:** Independent variable values of the process and their corresponding factor levels.

Independent variable	Symbols	Factor levels				
		-*α* (-1.682)	-1	0	+1	+*α* (1.682)
Temperature (°C)	A	16.6	20	25	30	33.4
Inoculum concentration (%, *v*/*v*)	B	6.6	8	10	12	13.4
Lantan camara fruit concentration (%, *v*/*v*)	C	6.6	8	10	12	13.4

**Table 3:** Uncertainties of the experimental measurements and total uncertainties for predicted values.

Parameter	Unit	Uncertainty value
*Experimental measurements*		
Uncertainty in the temperature measurement	°C	±0.5
Uncertainty in the weight measurement	g	±0.0021
*Predicted values (calibration equations)*		
Total uncertainty for residual sugar	Dimensionless	±0.68^a^
Total uncertainty for alcohol content	Dimensionless	±1.1^b^
Total uncertainty for total phenol content	Dimensionless	±4.3^c^

^a^Normal value was taken as 93.78. ^b^Normal value was taken as 9.55. ^c^Normal value was taken as 653.01.

**Table 4:** CCRD of three variables with the observed responses and predicted values for all responses.

Std	A Temp (°C)	B Ino.C. (%, *v*/*v*)	C L.F.C. (%, *v*/*v*)	Measured responses			Predicted responses		
				Al (%, *v*/*v*)	TP (mg L^−1^)	Sens.	Al (%, *v*/*v*)	TP (mg L^−1^)	Sens.
1	20 (-1)	8 (-1)	8 (-1)	6.7	352.8	5.9	6.17	392.19	6.12
2	30 (+1)	8 (-1)	8 (-1)	6.2	392.5	6.3	5.57	337.18	6.20
3	20 (-1)	12 (+1)	8 (-1)	8.4	301.6	6.1	7.10	263.07	7.65
4	30 (+1)	12 (+1)	8 (-1)	6.2	118.4	6	6.50	208.06	8.53
5	20 (-1)	8 (-1)	12 (+1)	7.3	400.7	6.5	7.18	381.99	5.19
6	30 (+1)	8 (-1)	12 (+1)	6.4	347.8	7.8	6.58	326.98	5.27
7	20 (-1)	12 (+1)	12 (+1)	8.4	467.4	6.4	8.12	481.08	6.72
8	30 (+1)	12 (+1)	12 (+1)	6.8	412.2	7	7.52	426.07	7.60
9	16.59 (-*α*)	10 (0)	10 (0	5.7	441.6	6.1	6.83	446.86	5.82
10	33.41 (+*α*)	10 (0)	10 (0)	6.3	367.9	6.7	5.82	354.34	6.62
11	25 (0)	6.63 (-*α*)	10 (0)	6.2	292.4	6.6	6.69	328.13	4.95
12	25 (0)	13.36 (+*α*)	10 (0)	8.1	346.9	6.9	8.26	302.87	8.19
13	25 (0)	10 (0)	6.63 (-*α*)	5.2	320.8	6.6	6.32	302.68	8.35
14	25 (0)	10 (0)	13.36 (+*α*)	8.5	467.6	8.9	8.03	477.42	6.78
15	25 (0)	10 (0)	10 (0)	9.9	669.8	8.9	9.44	643.07	8.88
16	25 (0)	10 (0)	10 (0)	9.9	670.6	8.8	9.44	643.07	8.88
17	25 (0)	10 (0)	10 (0)	9.5	632.9	8.9	9.44	643.07	8.88
18	25 (0)	10 (0)	10 (0)	9.8	661.8	8.9	9.44	643.07	8.88
19	25 (0)	10 (0)	10 (0)	8.5	531.7	8.7	9.44	643.07	8.88
20	25 (0)	10 (0)	10 (0)	9.1	690.3	8.9	9.44	643.07	8.88

Std, T, Ino.C., L.F.C., Al, TP, and Sens. represents to standard order, temperature, inoculum concentration, Lantana camara fruit concentration, alcohol, total phenol, and sensory, correspondingly.

**Table 5:** ANOVA evaluation of linear, interaction, and quadratic terms for alcohol, total phenol, and sensory response variables and coefficients of model prediction.

Source	Alcohol (%, *v*/*v*)					Total phenol (mg L^−1^)					Sensory analysis				
	DF	Coef.t	SS	*F* value	*p* value	DF	Coef.t	SS	*F* value	*p* value	DF	Coef.t	SS	*F* value	*p* value
Model	6	9.43	36.39	9.98	0.0003	7	643.09	4.220	20.26	<0.0001	7	8.86	26.69	41.53	<0.0001
*A-Temp.*	*1*	-0.31	*1.29*	*2.12*	*0.1695*	*1*	-27.50	*10327.15*	*3.47*	*0.0871*	*1*	0.23	*0.75*	*8.21*	*0.0142*
*B-InC.*	*1*	0.47	*2.99*	*4.93*	*0.0448*	*1*	-7.51	*769.94*	*0.26*	*0.6202*	*1*	-0.04	*0.018*	*0.20*	*0.6660*
*C-L.F.C.*	*1*	0.51	*3.54*	*5.82*	*0.0314*	7	51.97	*36879.35*	*12.39*	*0.0042*	*1*	0.53	*3.87*	*42.14*	*<0.0001*
*AB*	—	—	—	*—*	—	—	—	—	—	—	*1*	0.20	*0.32*	*3.49*	*0.0865*
*BC*	—	—	—	*—*	—	*1*	57.05	*26037.62*	*8.75*	*0.0120*	—	—			
*A* ^2^	*1*	-1.10	*17.54*	*28.86*	*0.0001*	*1*	-85.72	*1.059*	*35.59*	*<0.0001*	*1*	-0.94	*12.75*	*138.89*	*<0.0001*
*B* ^2^	*1*	-0.70	*6.99*	*11.51*	*0.0048*	*1*	-115.81	*1.933*	*64.95*	*<0.0001*	*1*	-0.82	*9.62*	*104.75*	*<0.0001*
*C* ^2^	*1*	-0.80	*9.29*	*15.28*	*0.0018*	*1*	-89.45	*1.153*	*38.75*	*<0.0001*	*1*	-0.46	*3.09*	*33.69*	*<0.0001*
Residual	13	—	7.90	—	—	12	*—*	35709.89	—	—	12	—	1.10	—	—
*Lack of fit*	*8*	—	*6.35*	*2.55*	*0.1586*	*7*		*19149.59*	*0.83*	*0.6062*	*7*	—	*1.07*	*21.77*	*0.18*
*Pure error*	*5*	—	*1.56*	*—*	—	*5*	*—*	*16560.29*	—	—	*5*	—	*0.035*	—	—
Cor total	19	—	44.29	*—*	—	19	*—*	4.577	—	—	19	—	27.79	—	—

Temp., InC., L.F.C., DF, Coef.t, and SS represent to temperature, inoculum concentration, Lantana camara fruit concentration, degree of freedom, coefficient, and sum of squares, correspondingly.

**Table 6:** Experimental data analysis for all predictive response models.

Statistical parameters	Responses		
	Alc (%, *v*/*v*)	TP (mg L^−1^)	Sensory
Std. dev.	0.78	54.55	0.30
Mean	7.65	444.39	7.35
C.V. (%)	10.18	12.28	4.12
PRESS	29.44	94241.42	4.89
*R* ^2^	0.8216	0.9220	0.9604
Adjusted *R*^2^	0.7393	0.8765	0.9372
Predicted *R*^2^	0.3352	0.7941	0.8242
Adequacy of precision	8.427	12.609	15.946
AAD (%)	0.950	2.696	1.169

Std. dev., C.V., Alc, TP, PRESS, and AAD stand for standard deviation, coefficient of variation, alcoholic content, total phenol content, predicted regression error sum of square, and absolute average deviation, respectively.

**Table 7:** Optimum values of fermentation variables and the predicted and experimental alcohol, total phenol content and sensory properties of the fermentation process.

Equations	Fermentation variables		Optimum values of variables	Optimum values of responses	
				Predicted	Experimental
Equation (4) Alcohol content (%, *v*/*v*)	Temperature (°C)	A	26.88	7.18	7.46
	Inoculum concentration (%, *v*/*v*)	B	10.94		
	Lantana camara fruit concentration (%, *v*/*v*)	C	10.17		
Equation (5) Total phenol (mg L^−1^)	Temperature (°C)	A	24.19	652.89	660.26
	Inoculum concentration (%, *v*/*v*)	B	10.09		
	Lantana camara fruit concentration (%, *v*/*v*)	C	10.61		
Equation (6) Sensory	Temperature (°C)	A	24.47	8.07	8.47
	Inoculum concentration (%, *v*/*v*)	B	11.4		
	Lantana camara fruit concentration (%, *v*/*v*)	C	8.67		

**(a):** 

Two − sided confidence = 95%; *n* = 3					
Factor	Name	Optimum level	Low level	High level	Coding
A	Temperature (°C)	24.80	16.59	33.41	Actual
B	Inoculum concentration (%, *v*/*v*)	10.16	6.63	13.36	Actual
C	Lantana camara fruit concentration (%, *v*/*v*)	10.65	6.63	13.36	Actual

**(b):** 

Response	Predicted mean	Predicted median	Std. dev.	*n*	SE Pred	95% PI low	Measured data mean	95% PI high
Alcohol (%, *v*/*v*)	9.55272	9.55272	0.846815	3	0.60	8.22	8.63	10.88
Total phenol (mg L^−1^)	653.007	653.007	54.0499	3	38.06	568.21	693.33	737.81
Sensory	8.90532	8.90532	0.290097	3	0.20	8.45	8.66	9.0

*n*, SE Pred, Std. dev., and PI represent number of confirmations, standard error of prediction, standard deviation, and predicted interval, respectively.

## Data Availability

The data and materials supporting the conclusions of this article are included in the article.
